# Multi-criteria optimisation problems for chemical engineering systems and algorithms for their solution based on fuzzy mathematical methods

**DOI:** 10.17179/excli2015-266

**Published:** 2015-08-26

**Authors:** B. B. Orazbayev, K. N. Orazbayeva, L. T. Kurmangaziyeva, V.E. Makhatova

**Affiliations:** 1 Atyrau Institute of Oil and Gas, Kazakhstan, Atyrau, microdistrict Sarykamys, Akchtanova street, house 20; E-mail: batyr_o@mail.ru; 2 Atyrau Institute of Oil and Gas, Kazakhstan, Atyrau, microdistrict Sarykamys, Akchtanova street, house 20; E-mail: kulman_o@mail.ru; 3 Atyrau State University. H. Dosmukhamedov, Kazakhstan, Atyrau, Azattyk avenue, house 72, flat 6 ( 060005); E-mail: Kurmangazieval@mail.ru; 4 Atyrau State University. H. Dosmukhamedov, Kazakhstan, Atyrau, Ondasynova street, house 8; E-mail: mahve@mail.ru

**Keywords:** multi-criteria optimisation, optimisation principles, benzene production, the task of fuzzy mathematical programming

## Abstract

Mathematical equations for the multi-criteria task of the optimisation of chemical engineering systems, for example for the optimisation of working regimes for industrial installations for benzene production, have been formulated and developed, and based on fuzzy mathematical methods, algorithms for their solution have been developed. Since the chemical engineering system, which is being researched, is characterised by multiple criteria and often functions in conditions of uncertainty, the presenting problem is formulated in the form of multi-criteria equations for fuzzy mathematical programming. New mathematical formulations for the problems being solved in a fuzzy environment and heuristic algorithms for their solution have been developed by the modification of various optimisation principles based on fuzzy mathematical methods.

## Introduction

Methods based on the use of fuzzy set theory (FST) (Dubois, 2011[3]; Kuzmin and Travkin, 1992[5]; Zadeh, 1965[23]; Rikov and Orazbayev, 1995[17]) are effective approaches to the optimisation of working regimes for complex chemical engineering systems (CES) in conditions of uncertainty arising from the fuzziness of input data. Technological systems in the petro-chemical and other industries are considered complex chemical engineering systems (CES), in which technological processes for the treatment of raw materials and products (for example benzene) are carried out, characterised by economic indicators, and during the functioning of which, the objects impact the environment, requiring that ecological production issues be addressed (Serikov and Orazbayeva, 2006[18]). Therefore, in order to optimise the working regimes of such CESs, it is necessary on the one hand, to solve the problem of the optimisation of economic and technological criteria (maximising profit, quantity and quality of the end product, improving technological indicators, minimising the cost price of the product and operational costs) and on the other hand to ensure the ecological safety of the production (maintaining ecological balance, minimising the risk of pollution etc.). For the efficient control of such objects, such criteria must have extreme values, that must be optimised. These problems are formulated in the form of multi-criteria optimisation equations (Orazbayev and Rikov, 1996[9]). 

Due to the large number and variety of the parameters, which determine the flow of the technological processes, due to the internal relationships between them and due to the actions of the operator which cannot be formulated mathematically, but which form an important and active element in the system for the control of the CES, the optimisation tasks are very complex. Furthermore, during the process of solving the task of optimisation of technological processes and the implementation of environmental protection measures, many problems arise in the CES in connection with the multitude of conflicting and fuzzily described criteria which determine the quality of the functioning of the system. In such cases, during the solving of the optimisation task, the basic data sources are people (specialist-experts, industrial staff, DM (Decision Maker), researcher in the sphere), that is his or her knowledge, experience, intuition and judgement which is expressed indeterminately and linguistically. 

In connection with the complexity or impossibility of measuring a range of parameters and indicators, many CESs and the technological processes which take place within them are difficult to describe quantitatively, which makes it difficult to use deterministic mathematical methods for the modelling and optimisation of their working regimes. This has led to the appearance of new methods for the formulation and solution of the problems in question, relying on fuzzy data, obtained from experts in the form of their judgements concerning the functioning of the object and taking into account their preferences in the process of optimisation and selection of solutions for the optimal control of the object (Rikov and Orazbayev, 1995[17]; Orlovskii, 1981[14]; Zaichenko, 1991[24]).

Methods to formalise and use such fuzzy data for the mathematical description of the functioning of the CES and for the solution of the optimisation problem and decision making in the process of their control, rely on expert procedures and the methodological theory of fuzzy sets. The successful solution of the modelling and optimisation problems for CESs which have been listed, requires the development of a methodology for the construction of fuzzy models for complex objects such as a CES, the further development of methods for the formulation and solution of the problem of optimisation of their working regimes in a fuzzy environment and the development of algorithms and programmes for the implementation of such methods using modern computer technology.

The various formulations, which are given in this paper, of the multi-criteria problem for optimisation based on different optimisation principles, modified for functioning in a fuzzy environment and the methods developed for their solution based on fuzzy data, are of current interest for the tasks of optimisation and control of CESs in industrial conditions. The research results for the theory are promising and widen the circle of practical problems which have been solved, allowing for the optimisation of complex CESs, taking into account their multi-criterial character and the fuzziness of input data. 

## Formulation of the Problems

**Formulation of the problems: **We formalise and formulate the equations for the optimisation of the parameters of the CES, for example technological installations in the oil industry in conditions having the problem of the multi-criteria nature of economic, ecological and technological characteristics and uncertainty arising from the fuzzy nature of available data. 

Let *f *=* f_1_*(*x*), ..., *f_m_*(*x*) the criteria vectors (objective functions), evaluating the results of the working of the technological objects being optimised, for example the economic efficiency and ecological safety of the technological objects. Each of *m *(*f_1_*(*x*), ..., *f_m_*(*x*)) local criteria depend on the vector *n* variables (control actions, regime parameters) *x *= (*x_1_*, ...,* x_n_*), for example: temperature and pressure and other parameters of the CES. We propose that this dependency is described by mathematical system models (Orazbayev and Rikov, 1995[8]; Orazbayev et al., 2014[7]). In practise, there are always various limits (economic, technological, ecological), which can be described by various limit functions


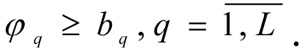


Variables *x *= (*x_1_*, ...,* x_n_*) also have their own variation intervals, determined by the process regulations of the CES and the requirements of environmental protection measures:


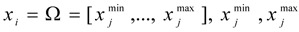


- lower and upper limits of the regime parameters *x_j_*. These limits may be fuzzy


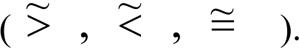


Values of the parameters *x *= (*x_1_*, ...,* x_n_*) must be chosen, which provide for the optimal working regime of the CES, that is ensuring the extreme values of the criteria vectors *f *=* f_1_*(*x*), ..., *f_m_*(*x*) while satisfying the given limits and with some fuzzy input data, and also taking into account the preferences of the person making decisions (DM).

**Mathematical formulation and models of the problems**: The problem which has been formulated, in conditions of multiple criteria and fuzziness, can be described in the form of the a multi-criteria fuzzy optimisation equation (fuzzy mathematical programming).

For the task of *fuzzy mathematical programming (FMP) *we understand the task contains an objective function or objective function vector (criteria, local criteria), which need to be optimised and system balanced or unbalanced, describing the conditions / limits, in connection with which a part or all of the elements of the equation (criteria, limits, information about their importance etc) are described fuzzily (Orazbayev and Rikov, 1996[9]; Orlovskii, 1981[14]).

The multi-criteria fuzzy optimisation tasks can be characterised by the following elements, the combination of which gives rise to specific optimisation tasks (Rikov and Orazbayev, 1995[16][17]) .

1. Criteria


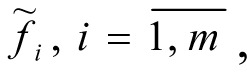


which are described in a fuzzy manner.

2. Instructions such as: «It is desirable that the value of


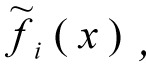


be large», that is the fuzzy operator of maximisation


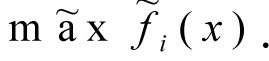


3. Fuzzy limits


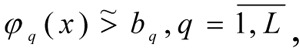


determining the admissible domain Ω of the multi-criteria tasks.

4. Information concerning the approximate importance of the criteria and limits (a range of priorities for local criteria *I_k_* = {1, ..., *m*}, and limits *I_r_* = {1, ..., *L*}, the weighting vector, reflecting the relative importance of the criteria (γ = (γ_1_, ..., γ_m_)) and limits (β = (β_1_, ..., β*_L_*)).

5. Fuzzy limits for the vector in the form of independent variables such as:


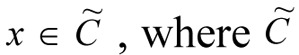


is a fuzzy set. 

6. Deterministic limits for the independent variable: x ∊ Ω.

In this way, the following sources of fuzziness can be distinguished: fuzziness of criteria, fuzziness of maximisation, fuzziness of limits (lack of a baseline set of alternatives and criteria), fuzziness of the relative importance of criteria and limits. Noting, that, for example, for the criteria limits, two sources of fuzziness can be immediately combined: fuzzy criteria and fuzzy instructions.

Taking into account the given information, the multi-criteria task for optimisation of the CES can be formulated as follows:

Find the optimal values of the vector variables *x *= (*x_1_*, ...,* x_n_*) that is


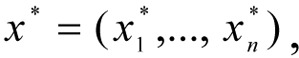


providing for such values of the local criteria, which satisfy the DM:


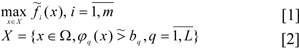


where


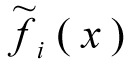


are the fuzzy local criteria, the values of which are calculated according to the model (some or all of which may be fuzzy) (Orazbayev et al., 2014[7]);


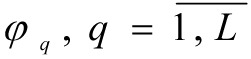


are the limit functions which determine the admissible domain Ω for the multi-criteria tasks [1]-[2]; *b_q_* are the given values, which may be fuzzy.

The solution of the given task are the values of the vectors for the regime parameters being optimised


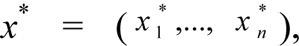


providing for such values of the local criteria which are optimum, that is satisfying the DM.

In the event that the criteria are fuzzy, then their membership function is maximised:


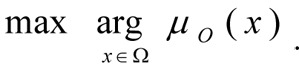


It is noted that the tasks [1]-[2] - happen to correspond when the productivity of the CES, the quantity and quality of product, profit etc. are chosen as the criteria. If the objective function [1] expresses loss, production expenses etc, then the maximisation tasks is transformed into a minimisation function, for example a negative objective function is introduced -


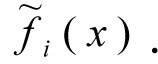


In the event of a variety of local criteria it is necessary to transform and normalise these criteria beforehand. 

In order to obtain a strictly mathematical formulation of the original problem, in the first instance it is necessary to consider the situation when the task of fuzzy mathematical programming is presented as a single criterium optimisation task (in the event of multi-criteriality, a contraction of local criteria to one integrate criterium can be carried out) and several limits, which are fuzzy. 

Let there be one normalised criterium - μ_0_(x) in the form of (1) and* L *limits in the form of (2) with fuzzy instructions -


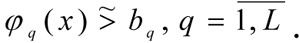


We are proposing that the membership function for the fulfilment of the limits


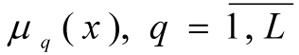


for each limit, can be constructed as a result of a conversation with the DM, specialists and experts. The main means for the reconstruction of this function is a graphic construction of the curved membership function of one or other parameter of the corresponding fuzzy set. Based on the resulting graph, a function type is chosen, which best approximates to it. After this, the parameters of the chosen function are identified. Practical experience of the construction of the membership function has shown that the membership function for fuzzy sets describing the given terms is sufficiently accurate in order to be able to approximate the exponential dependency (Orazbayev and Rikov, 1996[9]; Baronets and Grechikhin, 1992[2]; Orazbayeva, 2010[11]), for example in the form:


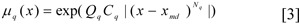


where μ*_q_*(x) - is the function (degree) of membership fulfilling the limit


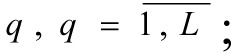


Q*_q_* - is the parameter which is found during the identification of the membership function and which determines the level of fuzziness; C*_q_*, N*_q_* - are the coefficients for the change in the domain for the determination of terms and the form of the graph of the membership function for fuzzy parameters; x*_md_* - is the fuzzy variable which best corresponds to the given term, for which; μ*_q_*(x*_md_*) = max μ*_q_*(x).

Given that the weighting vector β = (β_1_, ..., β*_L_*) reflecting the relative importance of the limits at the moment of formulation of the optimisation equation is known, then the fuzzy mathematical programming equation in its general form is:


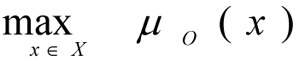


on the condition that


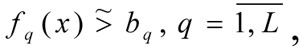


it can be written in the form:


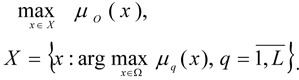


The given formulation of the fuzzy mathematical programming equation in the case of a non-fuzzy objective function and fuzzy limits with fuzzy instructions, reflects an attempt to maximise the objective function and completely satisfy the requirements of the limits. If, for example, all the membership functions are regular, then the formulation of the fuzzy mathematical programming has the form:


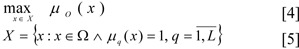


In this way, we have derived non-fuzzy mathematical programming [4]-[5] with a maximising of the objective function for the non-fuzzy set *Χ. *Further, we will propose a concavity of the objective function


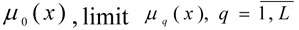


and convexity of the allowable set *Χ. *The given problem can be solved using ordinary methods and mathematical programming. 

## New Optimisation Tasks in a Fuzzy Environment and Algorithms for their Solution

In known formulations of the task of fuzzy optimisation and methods for their solution, generally, single criterium cases are considered, and there is no flexibility to take into account the preferences of the DM. Having said that, as a rule, the fuzzy task is transformed into its equivalent deterministic task at the stage of formulation, leading to the loss of a large part of the fuzzy input data which has been gathered (Zaichenko, 1991[24]; Yager, 1986[21]). 

In many cases, qualitative factors (fuzzy observations and judgements) are the main type of input data, as is habitual for human beings. The transformation of fuzzy descriptions into quantitative ones is not always successful or turns out meaningless. In this connection, the most promising approach is that which is based on a development of optimisation methods, adapted for human language, for qualitative factors of any kind, for human decision making procedures, for which the tasks are formulated and solved in a fuzzy environment, without being transformed into deterministic tasks, that is without loosing available data of a fuzzy nature. Recently, in scientific literature, papers have appeared which are dedicated to these approaches (Orazbayev and Rikov, 1996[9]; Aliyev et al., 1991[1]; Orazbayev, 2000[6]), which make use of modifications of various optimisation principles and compromise decision making schemes. In this paper, in order to solve the given problem, we have researched and proposed new optimisation principles and their combinations, which have been modified for working in a fuzzy environment. 

We bring equations [1] - [2] to the task of multi-criteria fuzzy optimisation, the main criteria for which are economic, ecological and technological indicators of a specific CES. 

Let


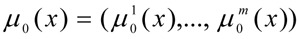


- be the normalised criteria vector,


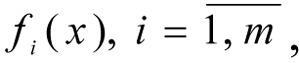


evaluating the efficiency of the working of the technological system for the production of benzene. Let each fuzzy limit


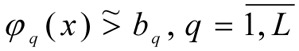


have a membership function constructed for its fulfilment


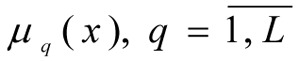


(for example using formula [3]). Any range of priorities for local criteria *I_k_* = {1, ..., *m*}, and limits *I_r_* = {1, ..., *L*}, or weighting vector, reflecting the relative importance of the criteria (γ = (γ_1_, ..., γ*_m_*)) and limits (β = (β_1_, ..., β*_L_*)) are known.

Then, modifying the idea of various *optimisation principles* for working in a fuzzy environment, then we can obtain various formulations of the multi-criteria fuzzy mathematical programming tasks and develop methods for their solution.

In practise, in the solution of actual optimisation tasks, it is often sufficient, that several principles are fulfilled with certain digressions. For such tasks of multi-criteria optimisation with several limits we are proposing that for the criteria, we use a new principle - the *principle of quasi-maxmin, *and for the limits the idea of a *method of ideal point*:


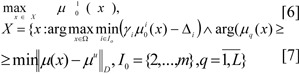


where ‖ ∙ ‖*_D_* - is the metric *D *being used, μ(x) = (μ_1_(x), ..., μ*_L_*(x)), μ*^u^* = (max μ_1_(x), ..., max μ*_L_*(x)). A possible variation is used as the coordinate for the ideal point μ*^u^* units: μ*^u^* = (1, ..., l)_._ Ω - is the input set determining the variables X_x_, *I*_0_ - is the set for criteria indices, taken to their limits.

In tasks [6]-[7] criteria with number 1 are maximised, and the rest of the criteria are taken to their limits using the quazi-maxmin principle (QMP), that is taking into account digression Δ*_i_*, fuzzy limits are reckoned based on a modification of the ideal point method (IPM).

### Algorithm QMP-IPM: 

1. In conversation with the DM, the value of importance coefficients for local criteria


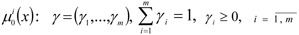


are determined.

^
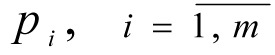
^

- the number of steps for each *i*-th coordinate is given.

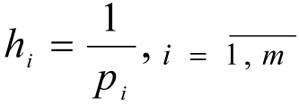

- the size of steps for changes to the coordinate of the weighting vector γ*_i_* is determined.

4. A set of weighting vectors γ^1^, γ^2^, ..., γ*^N^* is constructed, N = (*p*_1_+1)(*p*_2_+1)...(*p_m_*+1) is the variation in the coordinates at intervals of [0.1] with steps *h_i_*. 

5. The DM assigns the value of the digression from local criteria


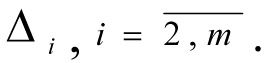


6. If


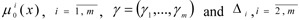


are fuzzy, then for these term sets are determined and a membership function is constructed.

7. The term set is determined and the membership function for the fulfilment of the limit


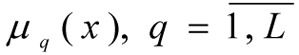


is constructed.

8. The coordinates of the ideal point are determined. The maximum values of the membership function - μ*^u^* = (max μ_1_(x), ..., max μ*_L_*(x)) can be used as coordinates of this point or units - μ*^u^* = (1, ..., l) (if the membership functions are regular).

9. The type of metric ‖μ(x) - μ*^u^*‖*_D_* is chosen, which determines the distance of the solution which is obtained x^*^ from the ideal point μ*^u^*.

10. The maximisation task


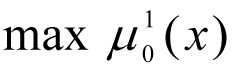


is solved for the set *Х*, determined by the expression [7]. The solutions


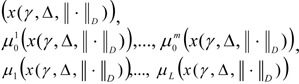


are determined.

11. The solution is shown to the DM. If the current results do not satisfy the DM, then he or she assigns new values for γ, and (or) Δ and (or) || ∙ ||*_D_* and it is necessary to return to step 2. Otherwise, continue to step 12.

12. The search for a solution comes to an end, and the results of the final choice are presented to the DM: the value of the control vector x^*^(γ, Δ, || ∙ ||*_D_*); the value of the local criteria


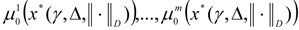


and degree of fulfilment of the limits


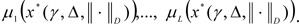


When steps 6 and 7 are being implemented, the membership function can be constructed using methods of expert evaluation (Orazbayeva, 2010[11]; Orazbayev et al. 2010[10]) and the formulae given above [3]. We give several variants of the use of Euclidean metrics (*D*=*Е*) for the implementation of step 9:


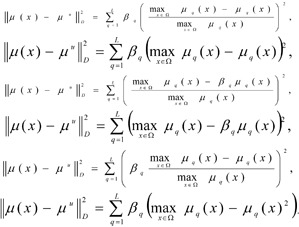


Let the range of priorities for the limit *I_r_* = {1, ..., *L*} be known. We set out the task of fuzzy mathematical programming, using and modifying the idea of the *main criteria method (MCM) *and the *lexicographic principle (LGP) of optimisation* (Pershin, 1994[15]):


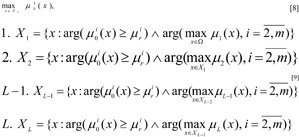


where the set *X_L_* is formed as a result of the solution of the sequence 1,2,…, *L, / *- is the logical symbol «AND», requiring that all the related values are true,


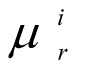


- are the limit values of the local criteria


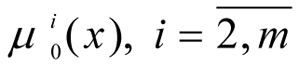


assigned by the DM. 

According to this schematic, initially the optimisation is carried out for the most important limit μ_1_(x) and a set is obtained for the optimal values of *X*_1_ for the criterium. Henceforth the criteria for the set *X*_1_ are optimised and a set of the optimal values for the second criterium *X*_2_ is obtained and so forth. 

Frequently, as a result of the solution of the first task, only a single point is obtained, and the solution of the problem comes to an end already at the first step, and the value of the second and following criteria are not taken into consideration. Insufficiencies in the lexicographic optimisation principle, connected to its “strictness” also become apparent here. It is possible to relax the strictness of the limit requirements, using the *lexicographic quazi-optimisation principle. *In that case the equations shown above [8]-[9] are rewritten as follows:


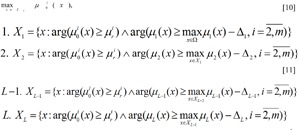


The dialogue algorithm for the solution of the optimisation tasks [8]-[9] and [10]-[11] is made up of the following main steps.

### Algorithm MC-LGP:

1. A range of priorities for the local criteria *I_k_* = {1, ..., *m*} is assigned (the main criterium should have priority 1).

2. The DM determines the limit values of the local criteria


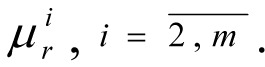


3. The term set is determined and a membership function is constructed for fulfilling the limits


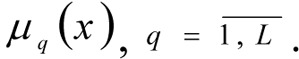


4. Based on information given by the DM, values are assigned to the digressions


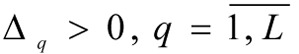


(in the event of use of the lexicographic quazi-optimisation principle, then in tasks [10]-[11] in the formulation of [8]-[9]


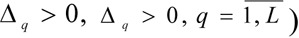


5. The task of maximisation of the main criterium


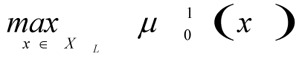


is solved for set *X_L_, *which determines the means of solution of the following tasks: 

in the arrangement of [8]-[9],


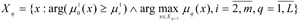


in the arrangement of [10]-[11]


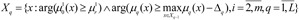


For *q* = 1: *X_q_*_-1 _= *X*_0_ = Ω.

Determine the current value of the solution, that is the values of: the control vector


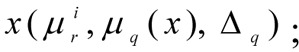


the local criteria


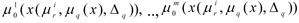


and degrees of fulfilment of the limits


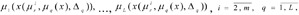


in the case of the formulation (8) - (9)


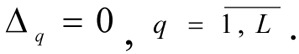


6. The solution is presented to the DM. If the current results to not satisfy the DM, then he or she assigns new values


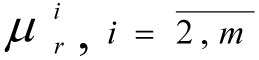


and (or) corrects the values μ*_q_*(x) and (or) Δ*_q_* (for formulation [10] -[11]


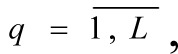


and return is made to step 3. Otherwise continue to step 7.

The search for a solution comes to an end and the results of the final decision are presented to the DM: the value of the control vector


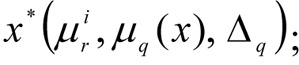


the values of local criteria


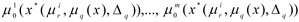


and the degree of fulfilment of the limits


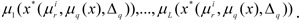


We come to the formulation of the multi-criterial task of fuzzy optimisation with a limit vector based on the *maxmin *principles (for criteria) and *Pareto *Optimisation (for limits):


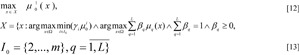


In the final task [12]-[13] the main criterium with priority 1 is maximised, the remaining criteria are taken to their limits using the maxmin principle, and the fuzzy limits are taken into account based on the Pareto optimisation principle (Pershin, 1994[15]).

The algorithm for the solution of this task has the following structure.

### Algorithm Max-Min Pareto Optimisation:

1. By means of conversation with the DM, the values of the weighting coefficients for the local criteria


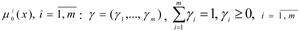


are determined.

2. If the criteria


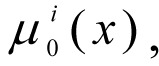


and the weighting vector γ are fuzzy, then for them term sets are determined and a membership function is constructed. 

3. By means of conversation with the DM, the values of the weighting coefficients for the limits


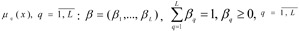


are determined.

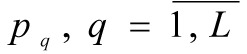

is assigned, the number of intervals for each *q*-th coordinate.

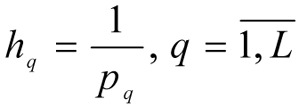

are determined, the values of the intervals for the changes to the coordinates of the weighting vector β*_q_*.

6. The set of weighting vectors is constructed β^1^, β^2^, ..., β*^N^*, *N* = (*p*_1_+1) ∙ (*p*_2_+1) ∙ ... ∙ (*p_L_*+1) variations in the co-ordinates with intervals of [0.1] and steps *h_q_*.

7. The term set is determined and the membership function for the fulfilment of the limits


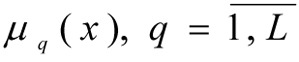


are determined.

8. Based on the model of the CES the maximisation task is solved


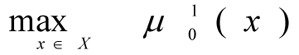


for set *Х*, determined by expression [13]. The current solution is determined


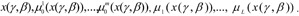


9. The solutions obtained are presented to the DM. If the current results do not satisfy the decision maker, then he or she assigns new values to, or corrects the values of γ and (of) β, and a return is made to step 2. Otherwise, continue to step 10.

10. The search for a solution comes to an end and the results of the final choice of DM are determined: variables x^*^(γ, β) providing for an optimal working regime of the CES; optimal values of local criteria


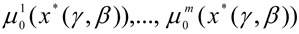


and a maximum degree of fulfilment of the fuzzy limits μ_1_(x^*^(γ, β)), ..., μ*_L_*(x^*^(γ, β)).

In such a way, the various optimisation tasks for the working regimes of the CES in a fuzzy environment are formulated. Based on various optimisation principles and methods using fuzzy set theory, the tasks have been arranged in the form of multi-criterial equations for fuzzy mathematical programming and heuristic algorithms proposed for their solution, which are one of the features of originality of this paper. Based on these results, it is possible to derive actual optimisation equations for the working regimes of various CESs.

## Practical Implementation and Discussion of Results

As an example of the implementation of the proposed approach to the solution of multi-criteria tasks for fuzzy optimisation, we consider the formulation of a mathematical arrangement and solution of tasks for the optimisation of the working regimes of technological installations for the production of benzene, which are functioning at the Atyrau Oil Refinery.

Using the research results which have been shown above, and based on a modification of max-min methods and the Pareto Optimisation Principle, the optimisation task for the optimisation of the benzene production process can be formulated and constructed in the following form: 

Let


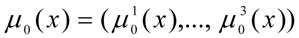


the normalised criteria, evaluating the product output from the technological installation for the production of benzene (benzene from the benzene column - 127÷138 thousand tons; refined petroleum from the benzene column - 77÷86 thousand tones; heavy aromatics from the rectification column - 445÷456 thousand tones). We propose that based on the information from the DM, experts and specialists, membership functions are constructed for the fulfilment of fuzzy limits


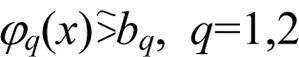


(the mean octane value of the benzene is no less 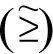 than 102; the sulphur content in the benzene is not more 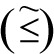 than 0.00005%) - μ_1_(x), μ_2_(x). The weighting vectors, expressing the relative importance of the criteria (γ = (γ_1_, ..., γ*_m_*)) and limits (β = β_1_, ..., β*_L_*)) are known.

Then the mathematical formulation of the task for multi-criteria fuzzy optimisation can be written in the following form:


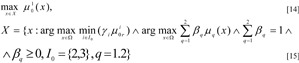


where ∧ - is the logical symbol «AND», requiring that all values connected with it are true,


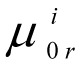


- are the limit values for the local criteria


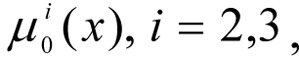


assigned by the DM. Changing the values of the weighting vectors for the criteria γ = (γ_1_, γ_2_, γ_3_) and limits β = (β_1_, β_2_) we obtained the solution to the task [14]-[15] - the values of the variables x^*^(γ, β), ensuring the maximum values of the criteria while not violating the limits, that is the optimisation of the working regimes of the object. The final decision is made by the DM, taking into account his or her preferences. 

In order to solve the equations which have been constructed for the optimisation of parameters for technological installations in benzene production [14]-[15] we used a modified combination of the *max-min methods and Pareto *Optimisation. Here the DM determines the weighting coefficients for the importance vector for local criteria and limits. Membership functions for the fulfilment of fuzzy limits are constructed based on expert evaluation with the help of an algorithm (Orazbayeva, 2010[11]) and formula [3] proposed in this paper.

We come to the specific Max-Min Pareto Optimisation algorithm for the solution of tasks [14]-[15] and the results obtained.

1. During the conversation with the DM, the values of weighting coefficients for the local criteria are determined


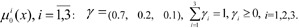


2. Since


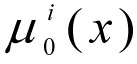


and γ are not fuzzy, it is not necessary to determine for them term sets or to construct a membership function. Criteria are normalised at [0.1] intervals and are determined using the models developed in these papers (Orazbayeva, 2009[12], 2010[13].

3. During the conversation the DM, experts and specialists, the values of weighting coefficients are determined for limits


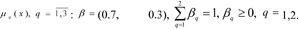

1. *p_q_*, *q* = 1,2 are determined - the number of steps, for each *q*-th coordinate: *p*_1_ = 5; *p*_2_ = 2.
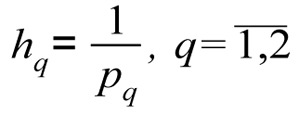

are determined - the size of steps for changes to the coordinates of the weighting vector


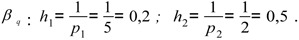


6. A set of weighting vectors is constructed β^1^, β^2^, *N* = (5+1)∙(2+1) = 18 the variation of coordinates at intervals of [0.1] with steps of *h_q_*, *q* = 1,2.

7. The term set is determined and membership functions are constructed for the fulfilment of limits μ*_q_*(x), _q_ = 1,2. The task being solved is described by two fuzzy limits: the mean octane value of the benzene 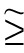 (is no less than) 102 and the sulphur content of the benzene 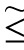 (is no more than) 0.00005%. In order to describe these fuzzy limits, the following set term has been determined: *T*(*X*.*Y*) = {low, below average, average, above average, high}. Using the given term set based on the formula [3] membership functions are constructed, describing the degree of fulfilment of fuzzy limits:


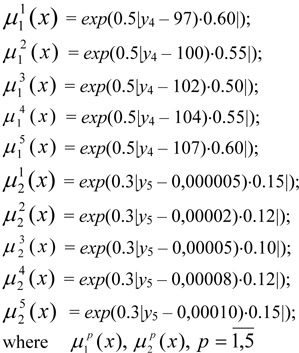


are the membership functions describing the degree of fulfilment of fuzzy limits for each quantum *р* for an average octane value of the benzene


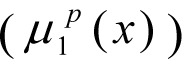


and sulphur content of the benzene


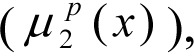


*y*_4_ and *y*_5_ - the numeric values of fuzzy indicators of the quality of the benzene, obtained on the basis of a level set α, the remaining coefficients having been considered and described in formula [3].

8. Based on the model of the CES describing the mutual dependencies of the local criteria and the variables (regimes parameters) x = (x_1_, x_2_, x_3_, x_4_, x_5_) (Orazbayeva, 2010[13]) the maximisation task is solved


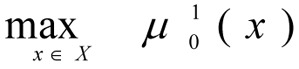


[14] for set *Х*, determined by expression [15]. Criteria are maximised for set *Х* taking into account the importance coefficients, determined in step 1. The current solutions are determined


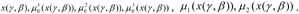


Here, it is possible to use a more appropriate method for the solution of the task which has been constructed, in our case the penalty function method which has been modified for use for working in a fuzzy environment. 

9. The results obtained are presented to the DM. If the current results do not satisfy the DM, then he or she assigns new values to, or corrects the values of γ and (or) β, and it is necessary to return to step 2; otherwise continues to step 10. In our example, the solution selected by the DM after 5 cycles are shown in Table 1(Tab. 1). 

10. The search for a solution comes to an end, and results of the final choice of the DM are determined: ensuring the optimal regimes of the CES (variables):


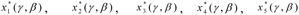


(see Table 1(Tab. 1)); optimal values of the local criteria


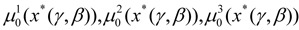


and a maximum degree of fulfilment of the fuzzy limits μ_1_(x^*^(γ, β)), μ_2_(x^*^(γ, β)) (see Table 1(Tab. 1)), x = (x_1_, x_2_, x_3_, x_4_, x_5_); γ = (γ_1_, γ_2_, γ_3_) = (0.7, 0.2, 0.1); β = (β_1_, β_2_) = (0.7, 0.3).

As a result of the analysis of the data which is shown in Table 1(Tab. 1) we come to the following conclusion:

1. The proposed Max-Min Pareto Optimisation* algorithm* makes it possible to solve the task with fuzzy limits without initially transforming them into deterministic variables, and, in comparison with the deterministic method, provides better results according to several indicators.
2. In the solution of the multi-criteria task with a fuzzy formulation, the adequacy of the solution of the production problems is improved, since fuzzy data is additionally used (knowledge and experience of industrial staff or specialists and experts), making it possible to adequately describe the actual situation without idealisation.
3. The Max-Min Pareto Optimisation algorithm makes it possible to determine the membership function, that is the degree of fulfilment of the fuzzy limits, ensuring that the task is solved with fuzzy limits, which are often found in industrial conditions.
4. During the optimisation process, with the help of the DM, it is possible to determine a solution which is a compromise between the quality and quantity of production.

In this way, so as to improve the quality of production (y_1_, y_2_, y_3_) in is necessary to reduce the volume. Therefore the formulation of the task for the maximisation of the output volume of benzene, with the simultaneous improvement of its quality is ill-defined. In this case, there may be two possibilities for a well-defined formulation of the task:

- The maximisation of the output product volume, while ensuring that qualitative indicators are no less than given values, that is the introduction and taking into consideration of limits for the product quality;
- The maximum improvement in the quality of the product, while ensuring a given output product volume, that is the introduction and taking into consideration of limits on the product volume.

The results shown in the table(Tab. 1) show the efficiency of the proposed algorithm for the solution of the fuzzy optimisation task, since, in comparison with the results of known methods (Shumskii and Ziryanova, 1981[19]; Yazenin, 1992[22]; Steuer, 1986[20]; Kahraman, 2008[4]), according to all the indicators, it gives results which are no worse, and for benzene and refined petroleum output, results which are better. In addition to this the Max-Min Pareto Optimisation algorithm makes it possible to take into consideration fuzzy limits and determine a degree of fulfilment of fuzzy limits. As can be seen, during optimisation, the complete fulfilment of fuzzy limits is ensured, that is their membership functions μ_1_(x^*^(β)) and μ_2_(x^*^(β)) are equal to 1. 

## Conclusion

New formulations for multi-criteria optimisation tasks for a CES, for example for a technological installation for benzene production, have been obtained, with ecological and economic criteria in the form of fuzzy mathematical programming tasks, and a set of heuristic algorithms has been developed in order to solve them. The algorithms, which have been developed, are based on a modification and combination of various optimisation principles (quazi max-min and ideal point, main criteria and lexicographic optimisation principles, max-min (guaranteed result) and Pareto optimisation). They have been modified for working in a fuzzy environment based on fuzzy mathematical methods.

The scientific originality of the results lies in the fact that the equations are formulated and solved in the fuzzy environment, without their initial transformation to their equivalent deterministic equations. In this way, in contrast to the approaches of other algorithms, in the proposed methods for the formulation and solution of multi-criteria fuzzy optimisation tasks, the fuzziness of the input data, obtained by means of the description of criteria and limits, is preserved, and, based on various optimisation principles, the problem of multi-criteria is solved in a form which is convenient to the DM. This provides for a more adequate description of the production situation in a fuzzy environment and efficient solutions of the optimisation tasks which arise can be obtained. Using these or other optimisation principles in the formulation of the task, we thereby give birth to various arrangements and solutions to the initial multi-criteria optimisation task, which allows the DM to exercise choice, and without a lot of thought he or she can compare them and choose the best and most convenient one according to his or her preference. 

The theoretical value of the paper lies in the development of the theory of vector optimisation in conditions of uncertainty and in the processing and development of optimisation methods in a fuzzy environment. The practical value of the paper is determined by the efficiency of the solution in conditions of multi-criteriality and fuzziness, which cannot be solved, or are difficult to solve using traditional mathematical methods. The results which have been obtained make it possible to efficiently solve complex industrial tasks and so raise the economic indicators and improve the ecological condition of the industrial installations. 

## Conflict of interest

The authors declare that they have no conflict of interest.

## Figures and Tables

**Table 1:**
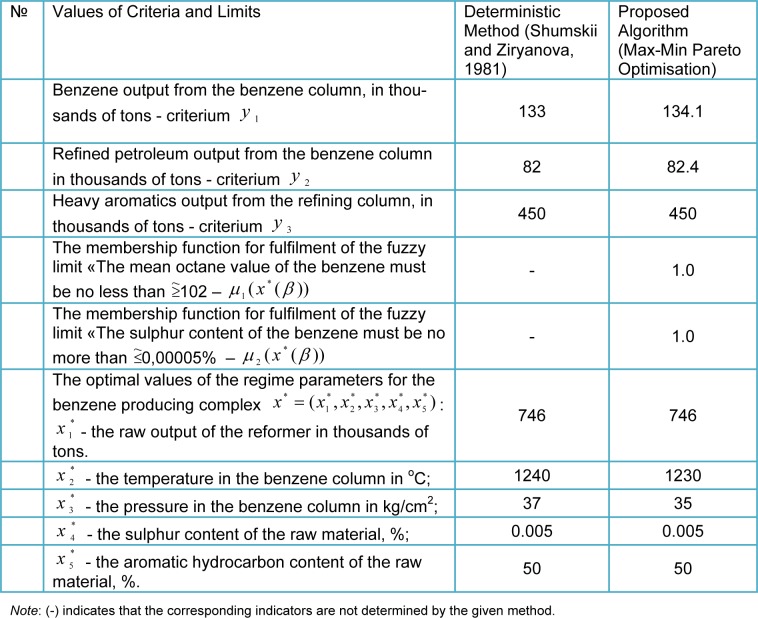
A comparison of the results of the solution of the optimisation problems using the proposed algorithm (Max-Min Pareto Optimisation), with those obtained using a deterministic method
